# Mesoappendix position variations in laparoscopic appendicectomy; a new anatomical classification to guide surgical strategy

**DOI:** 10.1007/s13304-025-02172-7

**Published:** 2025-03-21

**Authors:** Ernest Cheng, Raphael Shamavonian, Jasmine Mui, Zachary Bunjo, Amer Matar, Jon Barnard, Amit Sarkar, Wilson Petrushnko

**Affiliations:** 1 Department of Surgery, Coffs Harbour Health Campus, Coffs Harbour, NSW 2450 Australia; 2 George and Sutherland Hospital Clinical School, University of New South Wales, Sydney, NSW Australia; 3 Department of Surgery, The Tweed Hospital, Tweed Heads, NSW Australia; 4 Adelaide Medical School, University of Adelaide, Adelaide, SA Australia; 5 Innovation Surgical Teaching and Research Unit, Liverpool Hospital, Liverpool, NSW Australia

**Keywords:** Laparoscopic appendicectomy, Mesoappendix, Acute appendicitis

## Abstract

Dissection of the mesoappendix from the appendix is a crucial step in laparoscopic appendicectomies. Variation in the position of the mesoappendix during this common operation has not been previously described. We propose a classification system for the mesoappendix position seen laparoscopically and evaluate the impact each position has on operative difficulty and surgical approach. The mesoappendix positions in laparoscopic appendicectomies between January 2023 and January 2024 were classified into four categories from M1 to M4. Patients were grouped according to their mesoappendix positions. Outcomes evaluated included operative time, need for additional ports, use of energy devices, deviations from standard operative approach. Various mesoappendix positions were correlated with the intra-operative appendix position and histopathological findings. 104 laparoscopic appendicectomy cases were reviewed. 30 were classified as M1, 31 as M2, 27 as M3, and 16 as M4. Mean operative time was significantly longer for cases where the mesoappendix was in the M3 position (*p* > 0.001). This position was also more likely to require an additional port and deviate from the standard operative approach including need for retrograde dissection and staple cecectomy. We introduce a potentially surgical important classification system of the mesoappendix in laparoscopic appendicectomies. In this study, we attempt to validate the differences each position has on operative approaches and outcomes. We found that the M3 position is of greater difficulty to approach when dissecting the mesoappendix. This classification may serve as a valuable tool in guiding intra-operative surgical decision-making.

## Introduction

Acute appendicitis is a common presenting surgical emergency for which laparoscopic appendicectomy has become the established surgical standard of care [1]. As a common surgical procedure, it also serves in developing the foundation and initiation toward laparoscopic techniques for the trainee surgeon [2]. The operative complexity of this procedure depends on various factors, including appendix position, degree of inflammation, abdominal adhesions, perforation, and presence of an abscess [3, 4].

While the anatomical variation of the vermiform appendix in relation to with the caecum and terminal ileum is well-established [5, 6], there are few studies describing variations of the mesoappendix. The mesoappendix is derived from the layers of the ileal mesentery and contains the appendiceal branch of the ileocolic artery. Variability in the mesoappendix anatomy may result from congenital factors, degree of inflammation, peritoneal adhesions, gender, and age [5]. Previous evaluations of the mesoappendix have been largely in cadavers and open appendicectomies [6–9]. No studies to date have described the variations of the mesoappendix in laparoscopic surgery.

Variations in the mesoappendix are potentially important as a critical step in laparoscopic appendicectomy involves dissecting the mesoappendix from the appendix while controlling the appendiceal artery. There are several well-recognized approaches to this critical step utilizing a combination of electrocautery, energy devices, and laparoscopic clips [10]. In this study, we introduce a classification system to grade the variability in the mesoappendix position in the laparoscopic setting. We aim to validate its effectiveness by evaluating the impact of these positions on operative techniques and surgical outcomes during laparoscopic appendicectomies.

## Methods

**Fig. 1:**
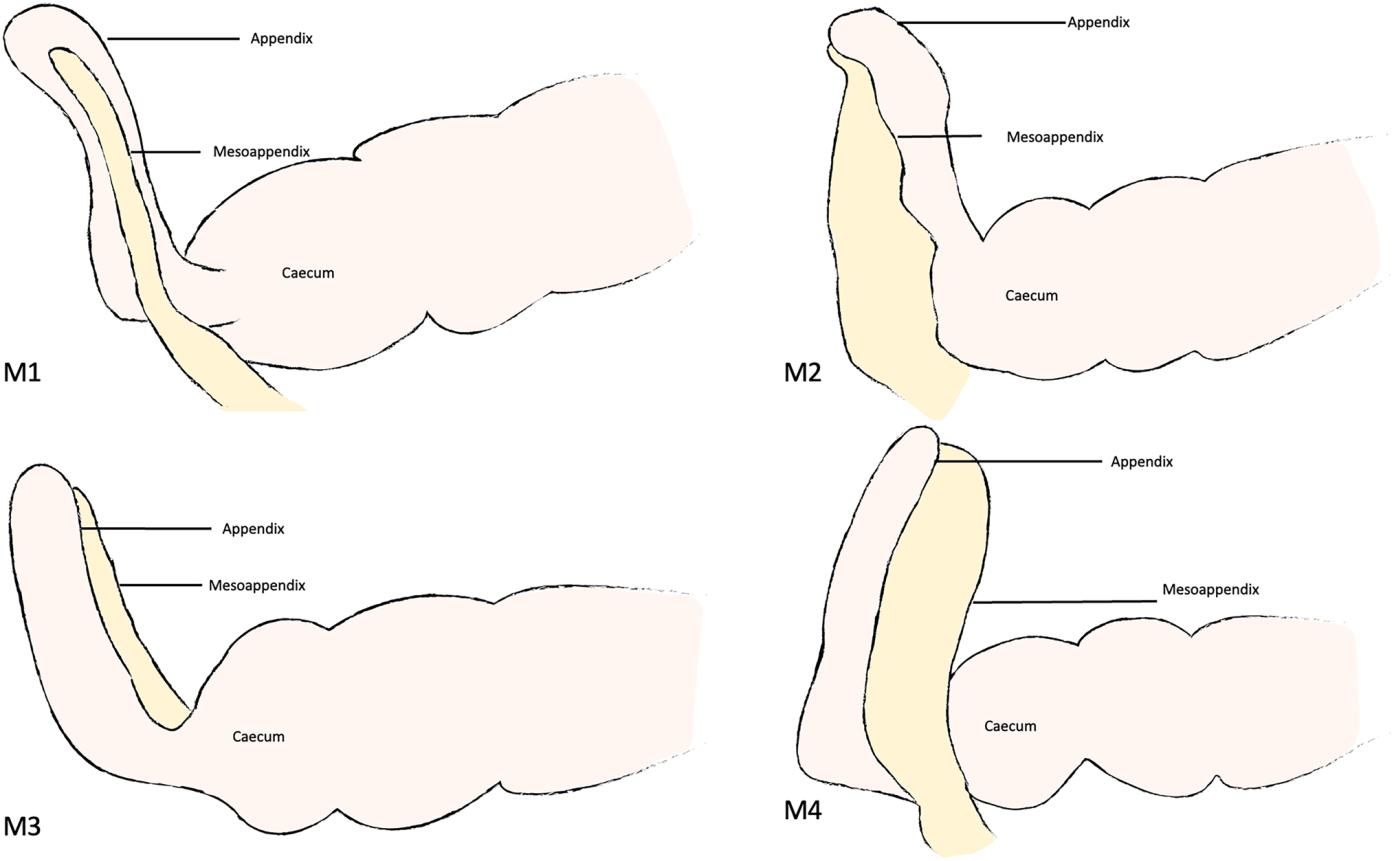
Schematic diagram of the four different mesoappendix positions

We systematically classified the mesoappendix into four distinct positions based on these principles. First, appendix and mesoappendix are freed from adhesions and are manipulated to the optimal position for dissection. In the laparoscopic view, the orientation in which the mesoappendix is facing and location relative to the appendix determines its position. Table 1 summarizes and provides a detailed description of each mesoappendix position and Fig. 1 illustrates these positions schematically.

**Table 1:** Summary of intra-operative classification of the mesoappendix position

Prior to classification	Mesoappendix description when viewed laparoscopically	Mesoappendix position classification
1. Appendix and mesoappendix is freed from adhesions and are manipulated to the optimal position for dissection 2. Orientation of mesoappendix is then determined	Mesoappendix is predominantly facing toward the laparoscopic camera	M1
	Mesoappendix is predominantly facing inferiorly toward the pelvis	M2
	Mesoappendix is predominantly facing toward the lateral abdominal wall away from the laparoscopic camera	M3
	Mesoappendix is predominantly facing superiorly toward the liver	M4

From January 2023 to January 2024, laparoscopic appendicectomies performed at Coffs Harbour Base Hospital were prospectively evaluated and the mesoappendix position classified. Our center is a 350-bed regional teaching health service in Australia with subspecialty general surgical services. We perform approximately 500 laparoscopic operations per year inclusive of emergency and elective procedures. This study was approved by the Local Health District’s ethics board. We excluded patients where the mesoappendix could not be clearly identified and open appendicectomies cases. Baseline demographic characteristics for all patients were extracted from the electronic medical records. Primary outcomes evaluated the incidence of each mesoappendix position in addition to operative factors including: appendix position, operative duration (defined as the time from skin incision to skin closure in minutes), insertion of intra-abdominal drains, need for additional laparoscopic working ports, use of energy devices apart from diathermy electrocautery, and any deviation from the standard surgical approach. Deviations were defined as instances requiring a retrograde dissection, conversion to open or more extensive resection). Secondary outcomes evaluated post-operative length of stay (LOS) recorded in days, post-operative complications according to the Clavien–Dindo classification [11], and histopathology results.

### Our standardized laparoscopic appendicectomy approach

A standardized three-port approach was used for all laparoscopic appendicectomy procedures with a 10 mm umbilical camera port and two additional 5 mm ports placed at the left lower quadrant and suprapubic area. A combination of graspers and hook diathermy is used to mobilize the appendix from inflammatory adhesions and facilitate exposure of the appendix and base of caecum.

Prior to the mesoappendix dissection, its position is evaluated and recorded according to our classification system. In most cases, dissection of the mesoappendix was initiated near the base of the appendix, with a double fenestrated grasper used to provide traction. The appendiceal artery was then carefully identified, dissected using a hook diathermy and secured with either clips or diathermy. Notably, in some cases, particularly those in the M3 position group, a varied approach was required. In these instances, dissection commenced either midway along the mesoappendix or near the tip, rather than at the base. Diathermy was then used to skeletonize the appendix from the mesoappendix down to the base. This variation was necessary when the base was not well-exposed or was difficult to access due to the mesoappendix's position.

Once the mesoappendix was separated from the appendix, the appendiceal base was then assessed. Endoscopic loop sutures were then placed at the base of the appendix prior to its removal. Additional procedures, such as peritoneal irrigation, drain placement, and more extensive resections, were determined based on operative indications.

### Statistical analysis

Analysis was conducted using IBM SPSS software version 24 (IBM corporation, New York, USA). Descriptive statistics were represented using mean, median, percentage, standard deviation (SD) and quartiles. Cross tables and chi-square tests were used to compare categorical covariates with Yates’ correction employed when appropriate. Continuous variables were compared using student t test or one-way analysis of variance (ANOVA). P value less than 0.05 was considered statistically significant.

## Results

A total of 104 patients underwent laparoscopic appendicectomy, with their mesoappendix position classified intraoperatively. No patients underwent upfront open appendicectomy and one patient could not have the mesoappendix position classified due to a phlegmon. The most common mesoappendix position classified was M2 with 31 cases (29.9%) followed by 30 cases of M1 (28.8%) and 27 cases of M3 (26%). The M4 position was observed in 16 patients (15.4%). The mean age was 35.9 years (SD 21.4), with the male-to-female ratio of 0.825 to 1. No difference was found between age (*p* = 0.590) and gender distribution (p 0.866) in all four groups. The mean American Society of Anesthesiologists physical status score (ASA) for all patients was 1.72 (SD 0.586). All surgeries were performed laparoscopically with no cases requiring conversion to open surgery.

Operative times for M1, M2, and M4 positions were similar; however, cases in the M3 position required significantly longer operative time. M3 cases had a median operative time of 70 min (range 50–84) compared to 43 min (range 31–52) for all other positions (*p* < 0.001). Additionally, M3 positions were more likely to require additional ports, with five cases requiring an additional laparoscopic working port compared to the other positions (*p* < 0.001). In contrast, neither M2 nor M4 positions required additional port insertions. There were no significant differences in the use of intra-abdominal drains between all four positions (*p* = 0.231). Three cases in the M3 position, as well as one case each from M1 and M2 positions, required the use of an energy device (LigaSure™) (*p* = 0.075). Laparoscopic appendicectomies performed with the M3 position present were more likely to deviate from the standard operative approach. This included three cases requiring retrograde or base-first dissection, where initial transection of the appendix was performed close to the base prior to separation from the mesoappendix. Four cases required staple cecectomies of which three were in the M3 position group. Table 2 summarizes these findings.

**Table 2:** Operative outcomes of each mesoappendix position

Position	*n* (%)	Median operative time, minutes (IQR)	Additional ports, *n*	Drain insertion, *n*	Energy device, *n*	Deviation from standard operative approach, n (approach)
M1	30 (28.8)	39 (26–58)	1	5	1	1 (retrograde dissection)
M2	31 (29.9)	45 (36–52)	0	5	1	1 (retrograde dissection) 1 (staple cecectomy)
M3	27 (26.0)	70 (50–84)	5	7	3	2 (staple cecectomy) 1 (right hemicolectomy) 2 (retrograde dissection) 1 (retrograde dissection and staple cecectomy)
M4	16 (15.4)	36.5 (30–49.5)	0	2	0	None
Total	104	47 (35–62.5)	6	19	5	9

*IQR* Interquartile Range

The most frequently observed appendix positions were retrocecal (59.6%) and pelvic (35.6). There were four subcecal and one pre-ileal appendix positions. Pelvic and retrocecal appendix positions were equally distributed among the four mesoappendix positions and comparative analysis revealed no correlation between the anatomical position of the appendix and the mesoappendix position (*p* = 0.51). The median post-operative hospital LOS was 2.10 days (range 1.20–2.95). Comparison between the mesoappendix positions and LOS revealed no significant difference (*p* = 0.118). Two post-operative complications were recorded and graded using the Clavien–Dindo classification for surgical complications [11]. One patient had a collection requiring interventional radiology drainage (Clavien–Dindo IIIb), while another experienced a post-operative superficial wound infection that required intravenous antibiotics (Clavien-Dindo II).

Histopathological analysis revealed no correlation between mesoappendix positions and the histopathological severity of appendicitis (*p* = 0.956) (Table 3). 87.4% of cases demonstrated acute suppurative appendicitis, 11.5% were complicated by either gangrenous, perforated, or abscess-associated appendicitis, and 1.9% showed a normal appendix. No malignancy was found in any of the cases.

**Table 3:** Histopathological analysis of each mesoappendix position

Position	*n* (%)	Normal appendix, *n* (%)	Acute suppurative appendicitis, *n* (%)	Gangrenous appendicitis, *n* (%)	Perforated appendicitis, *n* (%)	Peri-appendiceal abscess, *n* (%)
M1	30 (28.8)	1 (3.3)	27 (90.0)	1 (3.3)	1 (3.3)	0 (0)
M2	31 (29.9)	0 (0)	26 (83.8)	2 (6.5)	2 (6.5)	1 (3.2)
M3	27 (26.0)	1 (3.7)	23 (85.2)	1 (3.7)	2 (7.4)	1 (3.7)
M4	16 (15.4)	0 (0)	15 (93.8)	0 (0)	1 (6.2)	0 (0)

**Fig. 2:**
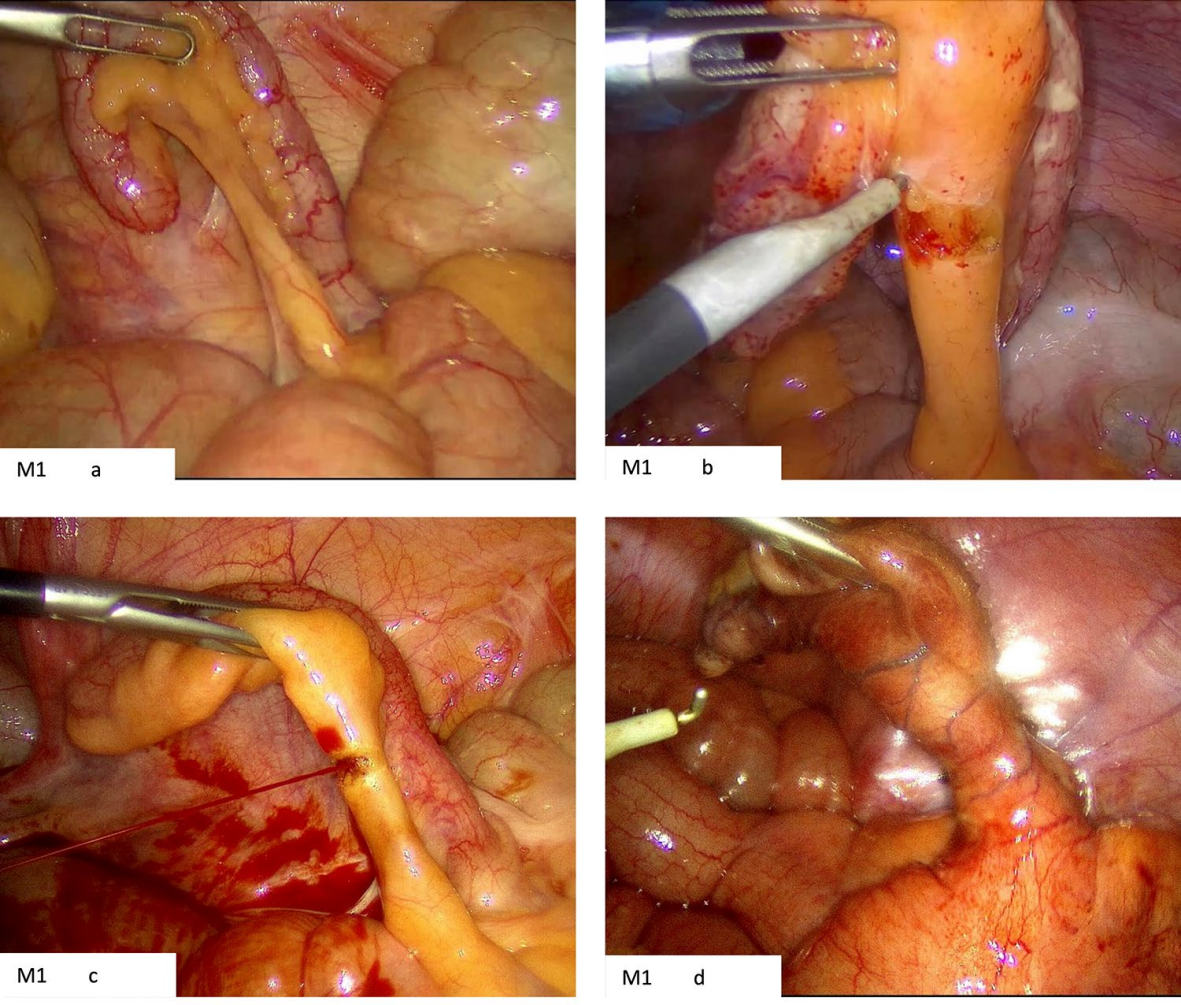
M1 position. **a** Clearly visualized mesoappendix with the appendiceal artery facing the camera. **b** Demonstrates the ease of accessibility of the appendiceal artery given the mesoappendix position. **c** Inadvertent injury to appendiceal artery but given its position it was easily controlled. **d** Inflamed mesoappendix in the M1 position

**Fig. 3:**
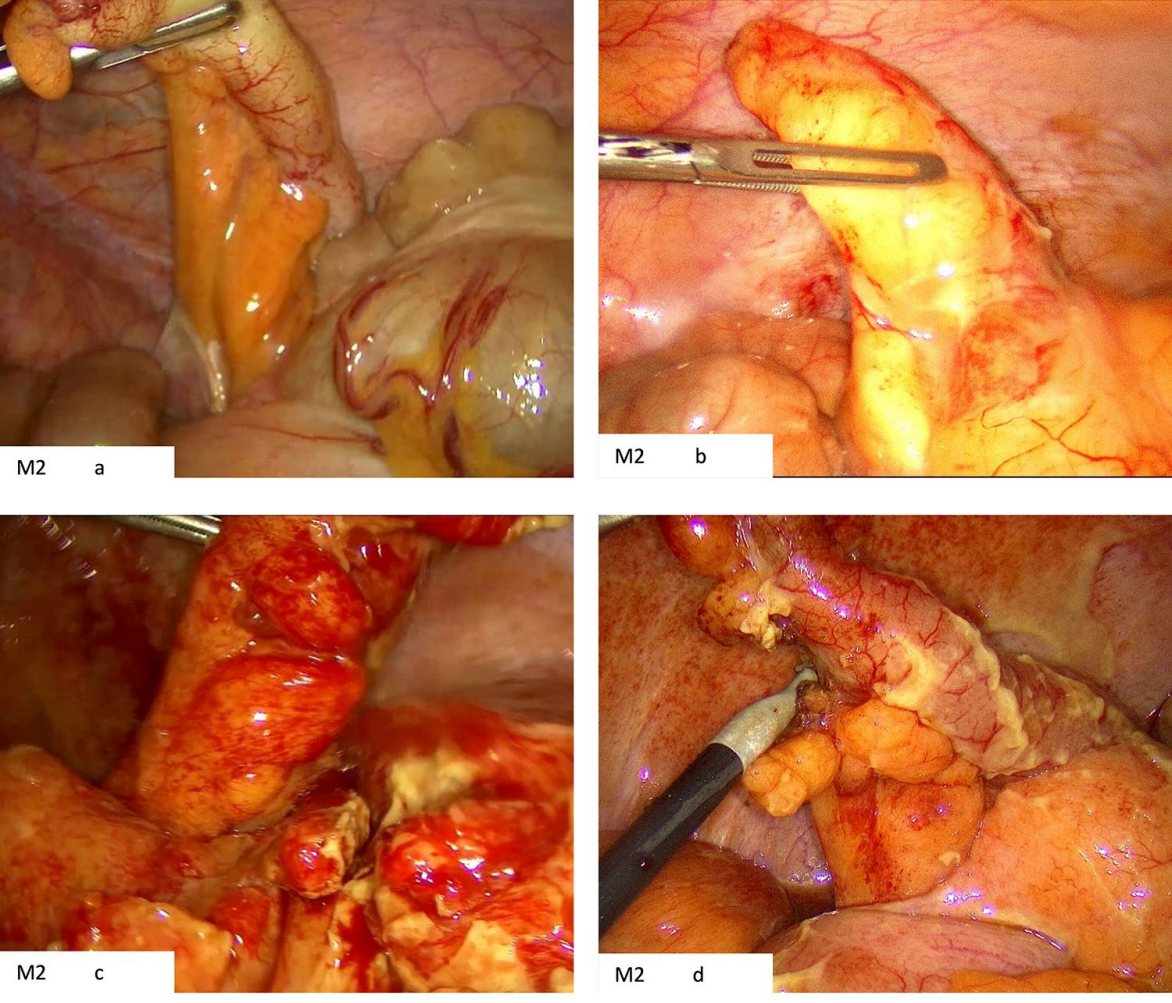
M2 position. **a** Demonstrates how the mesoappendix is “fanned out” toward the pelvis with providing access to dissect the appendiceal vessels. **b** Example of an inflamed mesoappendix in the M2 position. **c** Inflamed mesoappendix and surrounding tissues but due to the position, the dissection for this case was not difficult with an operative time of 35 min. **d** Example of the view between the appendix and mesoappendix in this position

**Fig. 4:**
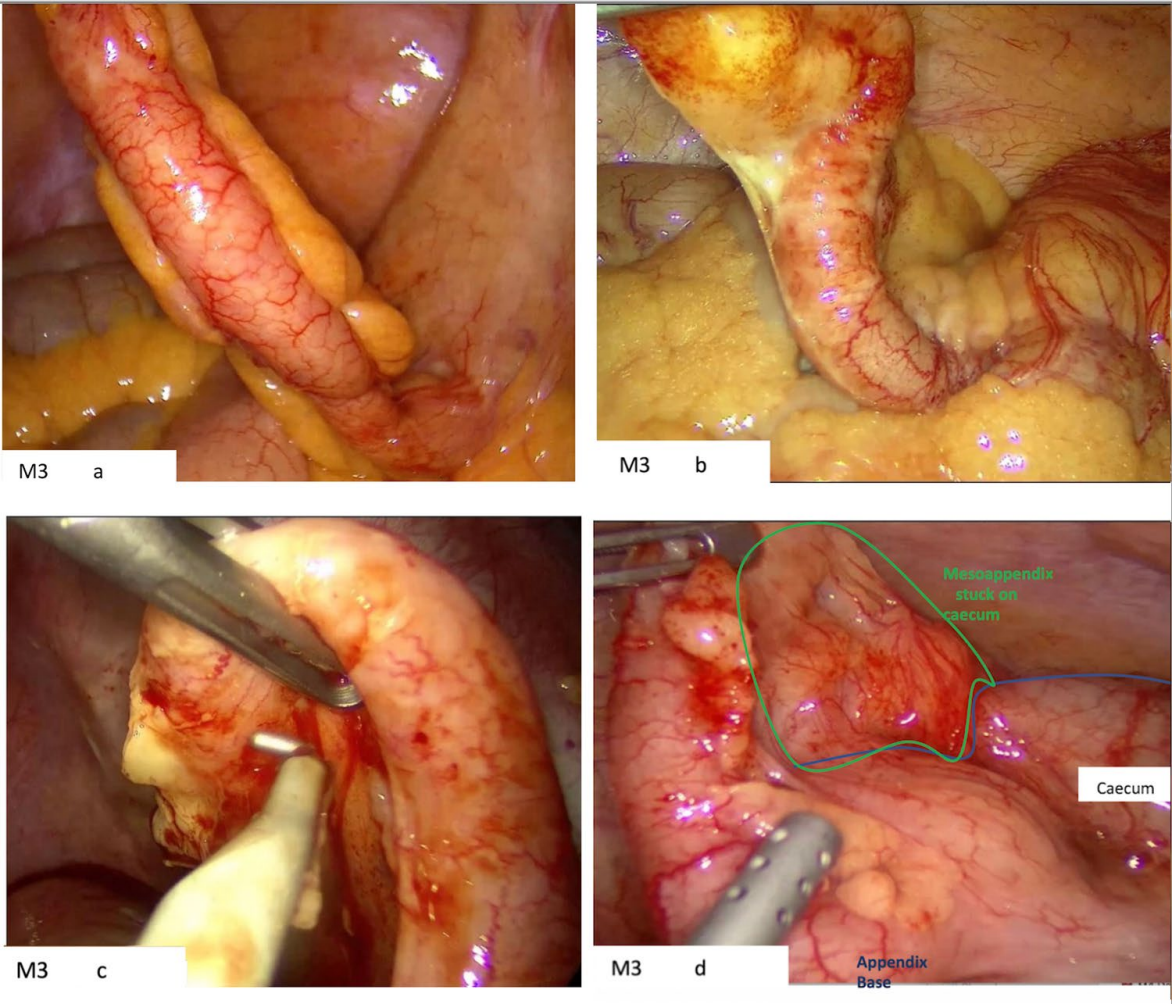
M3 Position. **a** The mesoappendix is facing away despite attempted manipulation of the grasper holding the appendix in several directions. **b** In this case, bleeding was encountered during dissection from the appendiceal artery. This was more difficult to control due to the mesoappendix position. **c** Limited exposure of the mesoappendix resulting in difficulty in dissection with the hook diathermy. Retrograde approach was used in this case. **d** Mesoappendix stuck on caecum in M3 position. An additional port was inserted in this case

**Fig. 5:**
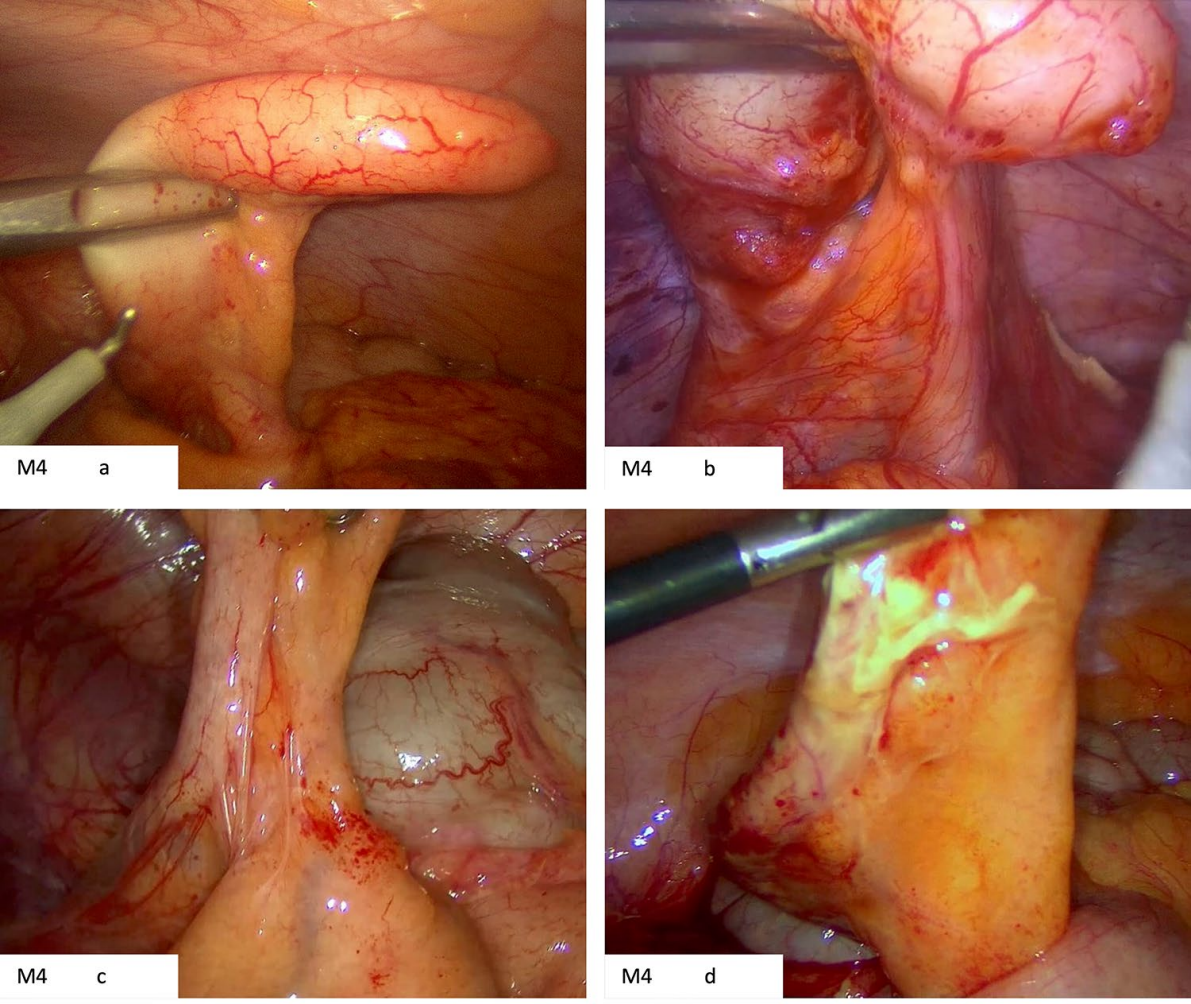
M4 Position. **a** Example of an M4 positioned mesoappendix. **b** Caecum is pulled up in this case demonstrating the risk of inadvertent cecal injury. **c** and **d** Further examples of the M4 mesoappendix position

Several examples were obtained from the laparoscopic cases to highlight the distinct characteristics of each mesoappendix position (Figs 2, 3, 4). Notably, Fig. 2c showcases the accessibility of controlling the appendiceal artery when in the M1 position, whereas Fig. 4c illustrates the challenge in controlling the artery when the mesoappendix in the M3 position. In Fig. 3c, a significantly inflamed and thickened mesoappendix is depicted; however, due to its M2 position, the dissection was straightforward, with an operative time of 35 min (Fig 5).

## Discussion

Our study introduces a novel classification system for the orientation of the mesoappendix during laparoscopic appendicectomies and attempts to validate its utility. We observed that variations in mesoappendix positions can significantly affect operative visibility and accessibility. Recognition of these variations may help predict complexity and inform the surgical approach needed to dissect the mesoappendix. Our results highlight the significance of the M3 mesoappendix position, which has potential implications for surgical planning and approach. Furthermore, we found no association between the position of the appendix and the histopathological results with the mesoappendix position. This study is first to describe these variations seen laparoscopically and their surgical implications.

Previous studies have described variations of the mesoappendix, but none have shown clinical significance, particularly in the operative setting. Mendez et al. described variations in the shape of the mesoappendix, with 68.2% being triangular and 31.8% oval [12]. Additional studies have also noted variations in the length and termination points where the mesoappendix ends on the appendix [6, 8, 12]. We hypothesize that these positional variations arise from combination these factors, along with the degree of inflammation, presence of adhesions, and anatomical variations in appendix and caecum.

Our results identified a distinctly challenging mesoappendix position (M3), where the mesoappendix faces away from the laparoscopic view. The appendiceal artery within the mesoappendix can be obscured by the appendix, making conventionally placed ports difficult to access. Early recognition of this mesoappendix position allows surgeons to consider alternative operative approaches to safely control the appendiceal artery and skeletonize the appendix. In our experience, achieving sufficient tension on the appendix in this position is challenging, limiting the effectiveness of diathermy during dissection. Considering an additional port, utilizing energy devices, or employing a retrograde approach may be optimal. Our results indicate that cases with an M3 position resulted in the greatest deviation from the standard operative approach compared to others. Utilizing these adjuncts and alternative approaches early in the procedure may facilitate a safer dissection and reduce operative time. Conversely, we found M1 and M2 positions to be more favorable overall. In these cases, the mesoappendix is triangulated by the two working ports, making the laparoscopic view and dissection of the appendiceal artery comparatively advantageous. In M4 positions, with the mesoappendix sitting along caecum, there can be a higher risk of inadvertent cecal injury.

Several studies have established grading systems for the laparoscopic assessment of appendicitis severity. Gomes et al. incorporated laparoscopic findings, such as degree of inflammation, necrosis location, abscess presence, and peritonitis into their grading system [13]. Similarly, the Sunshine Appendicitis Grading System utilized severity of inflammation and presence of purulent or feculent contamination to predict the risk of developing intra-abdominal collections [14]. However, both classification systems lacked consideration of how appendicitis grade influences the operative approach. Smigielski et al. proposed a novel tool to predict operative difficulty, incorporating factors, such as the quality of the appendix and mesoappendix. Their grading scale was specifically designed with the assessment of operative competency among trainees in mind [15]. Our study sought to establish a correlation between the mesoappendix position and the complexity of appendicitis (e.g., presence of abscess, gangrene, or perforation) based on histopathological findings; however, no significant correlation was identified. We hope future studies will evaluate the impact of mesoappendix positions on both appendicitis severity and operative difficulty.

We believe this classification of the mesoappendix position has strong relevance for surgical training, particularly those new to laparoscopic surgery. Classification of the mesoappendix position before dissection enables trainees and mentors to become cognizant to the challenges associated different positions. In our experience, trainees often attempt to dissect difficult M3 positioned mesoappendix, without considering a potentially safer retrograde approach. However, this method often requires close supervision as improper recognition of the base can lead to complications, such as the need for a cecectomy. Ultimately, by classifying mesoappendix positions, a standardized language can be established between trainees and surgeons, enhancing communication and improving surgical decision-making during operations.

This study explores a new classification system in a well-established procedure, but several limitations should be noted. As a pilot study, the relatively small sample size limits a comprehensive analysis without potential influence from random variability and selection bias. Additionally, we acknowledge that significant heterogeneity exists in operative approaches for laparoscopic appendicectomies, including the method of dissecting the mesoappendix. However, our surgical method is not unique, but a well-recognized and widely used cost-effective approach for performing laparoscopic appendicectomies. It is important to note our cases were conducted at a teaching hospital, where operative times may vary based on skill, experience, and the learning environment. Further analysis of the time taken specifically for mesoappendix dissection could provide added value to this study.

## Conclusion

The study is the first to develop and describe a novel system for categorizing the mesoappendix position in the laparoscopic setting. We identified distinct variations in the mesoappendix that may impact the surgical approach, complexity, and suitability for training purposes. Larger future studies are necessary to further validate the influence of the M3 position on operative techniques. Overall, this study introduces a classification system that could assist both surgeons and trainees in assessing case difficulty and in the early implementation of the most appropriate surgical approach.

## Data Availability

The data that support the findings of this study are available from the corresponding author upon reasonable request. Due to privacy and ethical considerations, access to the dataset may be subject to institutional approval.
